# Misdiagnosed pyriform sinus fistula revealed by iterative neck abscesses: A case report and review of the literature

**DOI:** 10.1016/j.amsu.2020.08.051

**Published:** 2020-09-09

**Authors:** Rabii Laababsi, Khadija Elbouhmadi, Anas Bouzbouz, Youssef Oukessou, Sami Rouadi, Reda Abada, Mohammed Roubal, Mohammed Mahtar

**Affiliations:** ENT Department, Face and Neck Surgery, Hospital August, 20’1953, University Hospital Centre IBN ROCHD, Casablanca, Morocco

**Keywords:** Abscess, Sinus fistula, Surgery

## Abstract

The pyriform sinus fistula is a rare condition described as an epithelialized tract connecting the skin of the neck to the foregut, and may result in cervical cysts and iterative abscesses misleading the diagnosis.

The clinical and radiological examinations are all useful. Surgery stands as one of the most effective therapeutic options consisting on the total excision on the eventual cyst, and the fistula that is followed to its inner opening on the pyriform sinus.

We present a case of a 3-years-old boy with a pyriform sinus fistula that caused recurrent neck abscesses treated independently delaying the diagnosis. Once in our structure, after radiological examination and antibiotics to cool the infection down, the surgery removed the cyst with its tract that opened in the pyriform sinus. The follow up showed an effective result with the total disappearance of the lesion with no more infectious episodes.

Even if it's a rare condition, the diagnosis of apyriform sinus fistula must be considered in front of every patient with a history of recurrentlatero cervical abscess.

## Highlights

• The pyriform sinus fistula is a rare condition described as an epithelialized tract connecting the skin of the neck to the foregut, and may result in cervical cysts and iterative abscesses misleading the diagnosis
• The clinical and radiological examinations are all useful
• Surgery stands as one of the most effective therapeutic options consisting on the total excision on the eventual cyst, and the fistula that is followed to its inner opening on the pyriform sinus

## 1 Introduction

The pyriform sinus « fistulae » are epithelialized tracts connecting the skin of the neck to the foregut [1], even though an external opening to the skin is rarely present [2]. Thus, the terms “pyriform fossa sinus”, “third or fourth branchial (or pharyngeal) pouch remnant”, or “third or fourth pharyngobranchial duct remnant” seem more appropriate [3]. These rare anomalies, arise from disturbances in the development of the fetalbranchialapparatus [1], and present with history of neck infections which delay the diagnosis [3].

## 2 Presentation of case

We present the case of a three years old boy, with no medical history, who consulted for a left cervical cystic lesion evolving during a year, with recurrent infection and abscesses requiring three hospitalizations in the pediatric department for IV antibiotics, Amoxicillin/Clavulanic acid 80mg/kg/day, associated with Metronidazole 50 mg/kg/day in 3 divided doses, for a week. Any improvement was observed besides the disappearance of the infectious signs. And, for the persistence of the lesion, and the recurrence of the abscesses, the patient ended up consulting in our structure.

The clinical examination found a left cervical soft cystic lesion, over the anterior border of the sternocleidomastoid muscle, with inflammatory tissues around, fistulated to the skin. With no other abnormality, normal tonsil lodges and no lymphadenopathies.

**Fig. 1:**
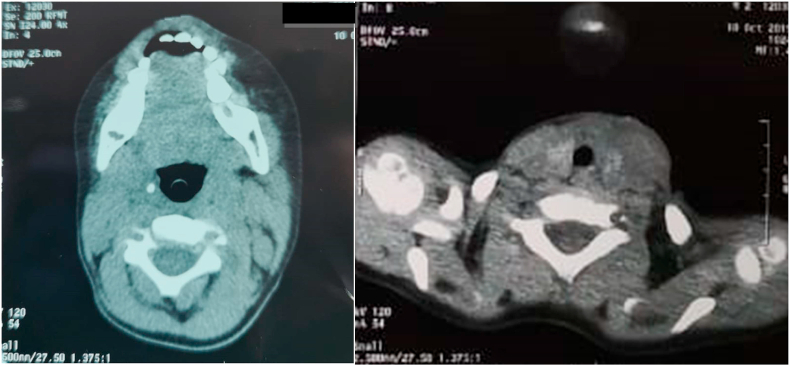
CT scan of our patient, showing the swelling of left cervical soft tissues. No gas was observed in the fistula tract nor within the thyroid.

On the computed tomography (CT) imaging, the lesion appeared as a thickening of the left cervical soft tissues. No cystic aspect nor septic collection nor air within the thyroid gland was identified (Fig. 1).

The patient was thus, programmed for an exploratory cervicotomy after seven days of antibiotics to cool the infection down, until the disappearance of the inflammatory skin signs.

**Fig. 2:**
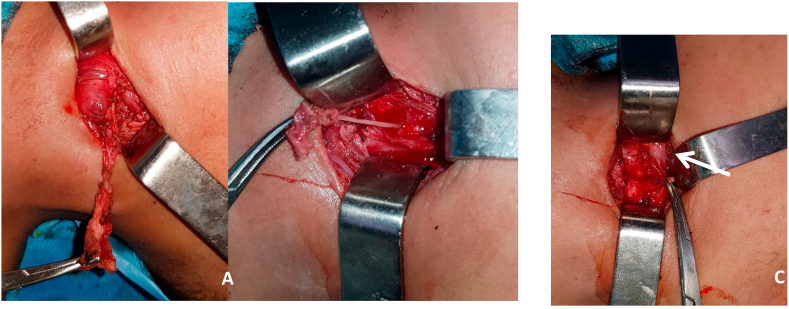
Surgical images showing the cyst and its fistula tract (A), the tract at its connexion to the pharynx (B), and the internal orifice at the pyriform sinus (white arrow) (C).

The surgery was performed under general anaesthesia, by a senior resident with 4 years of specialised training and consisted on an excision of the lesion following its tract, which passed posterior to the carotid artery, until the superior part of the thyroid gland, which appeared not to be affected and therefore was left intact as well as the recurrent laryngeal nerve and the superior laryngeal nerve. The thyroid cartilage and the inferior pharyngeal constrictor muscle were also spared. No anatomical relation was found between the tract and the hypoglossal nerve. And its trajectory never descended into the mediastinum. Finally, its sinus opening within the pyriform fossa was visualized (Fig. 2). No direct pharyngoscopy was performed.

The histological analysis of the operatory piece didn't find any skin appendagesnor cartilaginous residue confirming the ectodermic origin.

The follow up, on 8 months until today, showed a total disappearance of the cystic lesion with no more infectious episodes nor cervical abscesses.

This case has been reported in line with the SCARE 2018 criteria [4].

## 3 Discussion

### 3.1 Origin

The second, third and fourthbranchial ectodermic clefts form the cervical sinus of His which opens into the skin through one single orifice overlying the anterior border of the sternomastoid muscle, which is the location of the embryologic cervical sinus [1,5]. Thus, what can distinguish between their malformations is the localisation of the pharyngeal orifice. The fistulae of the thirdand fourth clefts open in the pyriform sinus, under the hyoid bone [5].

The classic description of thepathogenesisis based on the persistence of the pharyngobranchial duct, which connects the third and fourth pharyngeal pouches to the pharynx and normally degenerates during the seventh week of development, resulting in a sinus tract that communicates with the pyriform fossa [1].

### 3.2 Localisation

The course of the fistulae is located under the branchial derivatives of the associated arch and above the derivatives of the following one [5].

The thirdbranchial cleft fistula arises from the rostral end of the pyriform fossa [6] and would pierce the inferior constrictor medially to the greater cornuof the hyoid bone and above the superior laryngeal nerve. It would then loop over the hypoglossal nerve (nerve of the fourth arch), inferiorly to the glossopharyngeal nerve and pass posteriorly to the internal carotid artery. The fistula would then open in skin over the anterior border of the sternomastoid muscle. A thirdbranchial sinus or cyst might be found at any point along this course [3].

The fourthbranchialcleft fistula arises, lower than the third, from the pyriform sinus apex andfollows a more indirect route, from the pyriform fossa, medial and below to the superior laryngeal nerve, above the carotid bifurcation [1]. It then descends in the tracheoesophageal groove parallel to the recurrent laryngeal nerve into the mediastinum. It loops under the aortic arch on the left side and subclavian artery on the right (derivatives of the fourth arch arteries), follows the posterior side of the common carotid artery, and then exits at the skin surface [3].

### 3.3 Alternative theory for the embryologic origin

However, some clinical elements seem not to match with the previous established pathogenesis based on the direct communication across the branchial arch from pouch to cleft [1]. Indeed, unlike the second branchial fistulae, they very rarelypresent as a primary fistula. And even though the theoretical courses of their tracts are precisely defined in the literature, the surgical findings reported rarely match these descriptions [3].

To better fit the clinical presentation of these anomalies, James et al. proposed that theses fistulae derive from the thymopharyngeal duct, formed while the thymus descends during the seventh and eighth weeks, like the cysts formed along the thyroglossal tract while the thyroid gland descends from the tongue base to its final site. Besides, the simultaneity of these two events may explain that a failure of the thymopharyngeal duct to close would lead to the formation of a branchial sinus lined with endodermal cells arising from the piriform fossa and passing, in close association with the thyroid, toward the cervical inlet [2].

### 3.4 Epidemiology

The pyriform sinus fistulae are an extremely rare condition [7,8]. Described in all age groups, with a higher frequency in children and young adults, it also appeared that females are slightly more prone [1,7].

### 3.5 Clinical presentation

The clinical aspect varies with age. In the neonate, the condition is dangerous as the fistula can rapidly get enlarged with the infant swallowing saliva or milk, leading to tracheal compression, a stridor and respiratory distress [2].

In an older patient, the typical form ismore likely to appear as a laterocervical swelling, mostly described on the left side. Embryologically, the left fourth branchial arch artery forms part of the aortic arch, while the right one becomes the right subclavian artery. This normal asymmetry may be related to the noted left-sided predominance of the fistula, so the left-sided predominance may correspond to the fourth pouch origin of this condition [9].

The lesion is frequently exposed to infection causing recurrent lateral neck abscesses or cellulitis, as in our case and up to 42% according to literature [1], which is the main cause of a delayed diagnosis while the patient gets hospitalized multiple times for IV antibiotics or endures previous surgeries as incisions and drainages, whose sites may develop external sinus opening or pseudofistula. An acutesuppurative thyroiditis is also a common presentation wherepatients can be expected to be euthyroid [3].

The combination of a congenital sinus and acute suppurative thyroiditis was first described in the Japanese literature. Then, several similar cases have been reported subsequently, which are now believed to be infections arising from an underlying congenital pyriform sinus fistula, probably a remnant of the third or fourth branchial pouch [9].

In another hand, the noncommunicating or non infected communicating cysts may present as cold thyroid nodules. And the cysts that are partly or completely intrathyroidcan be confused with thyroglossal duct cysts [2].Also, the presence of a foreign body should suggest the diagnosis [7].

Other signs can possibly be associated such as dysphagia, odynophagia, sore throat, hoarseness, limitation of cervical extension, cough, upper respiratory infections, and even mediastinitisin a context of fever [2,7].

### 3.6 Imaging

According to the most common clinical presentation, the CT or magnetic resonance (MR) imaging is often the radiologic study of choice in delineating inflammatory lesions along the tract, even though the sinus or fistulous tract was not clinically apparent. With a superiority of the contrast-enhanced CT in detecting air density at the fistulous tract as well as in depicting thyroid gland involvement as a loss of high density in the affected gland, with a higher resolution [9].

The CT may reveal abnormal soft tissue swelling, an enhancing, often cystic mass associated with fat streaking and hypodensity within the ipsilateral (usually left) thyroid lobe [1,3].The pathway of the tract, if seen, is characteristic. It began at the pyriform sinus apex which appears deformed [1], coursed anteroinferiorly through the strap muscle layer, either beside or through the thyroid gland, and into the perithyroid space. And it appears enhanced with gas along its course [9].

Ultrasonography (US), even if limited in its ability to depict the hypopharyngeal lesions [9], can also be helpful. Showing a left-sided perithyroidhypo echoic area infiltrating the thyroid gland, as described by Hatabu and al in 1990 [10], while an image of gas within the area of the left upper pole of the thyroid gland is considered pathognomonic [1].

Barium esophagography can also be used to expose the hypopharyngeal fistulous opening. Its sensitivity varies, up to 50% [9] or even 80% [1]is some studies, but is still less useful in the acute inflammatory phase when the reactive oedema and inflammation may close the fistulous tract.

It is also interesting to observe the fistulous opening in the pyriform sinus through nasal fiberoptic endoscopy or through rigid pharyngoscopy or a direct laryngoscopy at the time of the surgery, even during acute episodes [3]. When the sinus tract is visualized, the CT fistulogram allowsdelineating the lesion course, which can prevent the use of unnecessary surgical exploration [1].

### 3.7 Treatment

The patient care should start by the administration of appropriate antibiotics to decrease the surrounding inflammation [1].

The surgical excision is the definitive treatment, performed several weeks after the infection has been resolved [2]. It is based on a direct cervical approach guided by the location of the cystic mass or a thyroidectomy approach [1,5]. It seems necessary to remove the cyst, its sinus tract with meticulous dissection, and the surrounding scar tissue to prevent recurrence [3].

The actual standard should add a hemithyroidectomy, if the gland is clearly affected. The main risk remains the recurrent laryngeal nerve, intimately associated with the tract, and often surrounded by fibrotic tissue resulting from chronic thyroiditis and recurrent abscesses [3]. Attention should also be given to the superior laryngeal nerve, at the thyroid-hyoid membrane level [5].

To ease the localisation on the sinus opening, an internal or external catheterism of the fistula can be done with a Fogarty catheter [1].Is also described in the literature, the use of methylene blue, but its extravasation makes the identification of important structures such as the recurrent laryngeal nerve difficult [3].

Chemocauterization or electrocauterization are less invasive, but less efficient alternatives with a higher rate of recurrence. The chemocauterization with 40% trichloroacetic acid (TCA) with or without primary mucosal coverage allows the obliteration of a longer segment of the fistulous tract depending on the deep of the acid penetration, with less risk to adjacent structures from heat and electrical spread and it can be safely repeated [1].

## 4 Conclusion

The pyriform sinus fistula is a rare condition but must be considered in front of every patient with a history of recurrent cervical abscess, in either side of the neck, in any child or young adult.

Also, it has become accepted that thyroid abscesses in children often indicate an underlying branchial remnant, especially when cultures reveal a mixed flora [1,2].

The surgical treatment appears to be the most effective in order to cure the condition, with a satisfying postoperative follow-up.

We presented a case report that supports the literature data concerning the clinical and therapeutic aspects of the pyriform sinus fistula.

## Ethical approval

This work has been approved by the ethical committee of our department.

The parents of the young patient (3 years old) gave their written informed consent for the surgery and the follow up.

## Funding sources

This research did not receive any grant or funding from governmental or private sectors.

## Author contribution

Rabii Laababsi: Corresponding author writing the paper.

Khadija El Bouhmadi: Writing the paper.

Sami Rouadi: Study concept.

Redallah Larbi Abada: Study concept.

Mohamed Roubal: Correction of the paper.

Mohamed Mahtar: Correction of the paper.

## Registration of research studies

Name of the registry: Research registry.

Unique identifying number or registration ID: 5848.

Hyperlink to your specific registration (must be publicly accessible and will be checked): https://www.researchregistry.com/browse-the-registry#home/registrationdetails/5f2028117a1cfd0016771167/

## Guarantor

Rabii Laababsi.

## Informed consent

The patient's parents gave informed consent for publication.

## Provenance and peer review

Not commissioned, externally peer reviewed.

## Declaration of competing interest

All authors have no conflict of interest or financial support with this article.
